# Trained immunity: a cutting edge approach for designing novel vaccines against parasitic diseases?

**DOI:** 10.3389/fimmu.2023.1252554

**Published:** 2023-10-06

**Authors:** Jinhang Zhu, Jiaxi Liu, Chao Yan, Dahui Wang, Wei Pan

**Affiliations:** ^1^ Jiangsu Key Laboratory of Immunity and Metabolism, Jiangsu International Key Laboratory of Immunity and Metabolism, Department of Pathogen Biology and Immunology, Xuzhou Medical University, Xuzhou, Jiangsu, China; ^2^ The Second Clinical Medical College, Xuzhou Medical University, Xuzhou, Jiangsu, China; ^3^ Liangshan College (Li Shui) China, Lishui University, Lishui, Zhejiang, China

**Keywords:** parasitic diseases, vaccine, trained immunity, innate immune memory, metabolic and epigenetic programming

## Abstract

The preventive situation of parasitosis, a global public health burden especially for developing countries, is not looking that good. Similar to other infections, vaccines would be the best choice for preventing and controlling parasitic infection. However, ideal antigenic molecules for vaccine development have not been identified so far, resulting from the complicated life history and enormous genomes of the parasites. Furthermore, the suppression or down-regulation of anti-infectious immunity mediated by the parasites or their derived molecules can compromise the effect of parasitic vaccines. Comparing the early immune profiles of several parasites in the permissive and non-permissive hosts, a robust innate immune response is proposed to be a critical event to eliminate the parasites. Therefore, enhancing innate immunity may be essential for designing novel and effective parasitic vaccines. The newly emerging trained immunity (also termed innate immune memory) has been increasingly recognized to provide a novel perspective for vaccine development targeting innate immunity. This article reviews the current status of parasitic vaccines and anti-infectious immunity, as well as the conception, characteristics, and mechanisms of trained immunity and its research progress in Parasitology, highlighting the possible consideration of trained immunity in designing novel vaccines against parasitic diseases.

## Introduction

As one of the most entrenched pathogens in the world, parasites have been posing severe threats to both individual health and the social economy for a long history. In 2018, malaria accounted for an estimated 228 million cases and 405,000 deaths globally, with even more people at considerable risk. According to the report of the World Health Organization (WHO), 11 parasitic diseases have been listed as Neglected Tropical Diseases (NTD) that infect over 1 billion people worldwide (1). Parasitic diseases have been threatening human health profoundly, causing physical pain and disability of substantial populations, especially in marginalized areas with poor sanitation and a backward economy. Therefore, reasonable solutions to control the global burden of parasitic diseases make great significance in individual and social welfare by improving health conditions, promoting productivity, slashing medical expenses, and alleviating general poverty.

Generally, the prevention and control strategies for parasitic diseases mainly rely on eliminating infection sources, blocking transmission routes, and protecting susceptible populations. Drug treatment has been the fundamental therapeutic avenue for quite some time. However, accumulating studies have reported drug resistance in both ecto- and endo-parasites, which undoubtedly impedes curing parasitic diseases (2–4). Other preventive measures (*e.g.*, fecal management, water purification, pesticide sprays, intermediate host elimination) are labor and time-consuming, as well as financial resources. With the development of modern technology, a series of new avenues have been employed to improve efficiency (5–7). However, wide hygiene-based measures are crucial for the fight against parasitic diseases, and additional measures are needed to bring them under control.

Featuring simplicity, effectiveness, and long-term preventive capability, vaccines are undoubtedly a vital tool for controlling infectious diseases on a large scale and compensate for the deficiency of drug and surgical treatment to a large extent. Historically, the success of smallpox and polio vaccines has enabled us to eliminate these diseases. However, it is frustrating that no commercialized parasitic vaccine is available for humans, merely with several vaccines under different stages of clinical trials. RTS, S/AS01 (Mosquirix, GlaxoSmithKline) is the best example of a parasitic vaccine, the only malaria vaccine in the phase 3 clinical trial. It is reported that within 12 months after vaccination, the vaccine can halve the parasitic incidence in children aged 5 - 17 months and provide protection against severe malaria for children in Africa (8). However, the protective efficacy is only 28.3% among infants who received three doses and 36.3% for those given a fourth dose (9). Notably, a follow-up study over 7 years shows the vaccine efficacy wanes significantly over time, with 3.6% at year 7 in the low-exposure cohort and a negative rate at year 5 in the high-exposure cohort (10). Based on recent findings, a new generation of vaccine candidates has been developed, which contains antigenic domains similar to the RTS, S vaccine and can be expressed on mRNA or nanoparticles. According to the latest human trials in Burkina Faso with children injected with the nanoparticle R21 vaccine, the initial results showed 77% protection against severe malaria 1 year after 3 doses of the vaccine in children (11). Another example is the current status of the schistosomiasis vaccine. *S. haematobium* 28-kD glutathione S-transferase (rSh28GST), the first vaccine to enter a clinical trial for urinary schistosomiasis, has failed due to its lower safety and immunogenicity (12). Moreover, another three candidates (*S. mansoni* 14-kDa cytoplasmic fatty acid-binding protein, Sm14; *S. mansoni* 9-kDa tetraspanin surface protein, Sm-TSP-2; *S. mansoni* surface calpain, Sm-p80) are now under varying stages of clinical trials, of which the protective efficacy is unavailable and still needs further estimation (13–15).

With the development of research, several novel vaccines have emerged as a hot spot in parasitic vaccine development. Intriguingly, DNA vaccines effectively induce cell-mediated Th1 immune responses with increased proliferation of CD4^+^ T cells and CD8^+^ T cells in animals, and have a higher safety profile than vector vaccines (16–23). Additionally, DNA vaccines have a superior performance in terms of stability at ambient temperatures, rapid adaptation to new targets, and cost-effectiveness (18, 24). For example, the DNA vaccine pVIVO2-Sj23.EGFP against *S. japonicum* has a systemic and long-lasting protective effect and is safe for gut microbiota in mice (25). In the preclinical context, DNA vaccines have been developed against *Schistosoma* spp (26–29)., hookworms (30), and *Fasciola hepatica* (31). Unfortunately, many of these vaccines exhibit little reduction in parasite loads. Recently, the recombinant protein enhancing Sm-p80 DNA vaccine with CpG dinucleotides as an adjuvant, shows the most significant protective effect, reducing the parasite burden of *S. mansoni* by 47.34% (32). Moreover, viral vectored vaccines use infectious agents to deliver the vaccine target, leading to a potent cellular immune response, particularly CD8^+^ cytotoxic T cells (16). What’s more, viral vectored vaccines can mimic natural infections and are highly immunogenic (33). It is encouraging to note that viral vectored vaccines have been developed against malaria (34), *Echinococcus granulosus* (35) and taeniasis (36).

Despite the inability of the human host to develop a naturally acquired immune response to hookworms, it is feasible to develop a hookworm vaccine based on the success of immunizing experimental animals with an attenuated larval vaccine or with antigens extracted from the alimentary canal of adult blood-feeding stages (37). However, only recombinant proteins produced in eukaryotic expression systems (*e.g.*, insect cells, baculoviruses, and yeast) are immunologically similar to the corresponding natural antigens (37). A challenge for vaccinologists is to formulate selected eukaryotic antigens with suitable adjuvants to induce the production of high litres of antibodies. Furthermore, veterinary vaccines can reduce disease prevalence in humans and animals. For example, Listeria monocytogenes vaccines expressing the tachyzoite surface antigen NcSAG1 (Lm3Dx_SAG1) plus kinase inhibitor BKI-1748, apical membrane antigen 1 (AMA-1) vaccine candidate, Trichobovis vaccine and *Toxoplasma. gondii* bradyzoite-formation deficient 1 (TgBFD1) vaccine candidate have all made significant progress in animals (38).

Insufficient efficacy is the major obstacle to the success of human parasite vaccines. For the unique biological characteristics of parasites, parasitic genomes, antigenic components, and life cycles are of more complexity and diversity in contrast with bacteria and viruses. Parasites express specific antigens and elicit differential host responses at different life stages. As a result, most parasitic antigens only induce partial protective immunity, which undoubtedly aggravates the difficulty in finding a qualified vaccine candidate to induce long-term, specific, and effective immune responses (39). On the other hand, many parasites utilize advanced strategies to regulate the host’s metabolism and immunity (40), compromising parasitic vaccines’ effect (41). Considering the status of the current parasitic vaccine, it is, therefore, essential to boost vaccines’ protective effect by other pathways. Trained Immunity (TI), also termed Innate Immune Memory, has attracted our interest because the novel discipline has been increasingly recognized to provide a particular perspective for vaccine development targeting innate immunity (42). Herein, we reviewed the anti-innate immunity of parasitic infection in hosts, as well as the conception, characteristics, and mechanisms of trained immunity and its advance in the field of Parasitology, highlighting the possible consideration of trained immunity in designing novel vaccines against parasitic diseases.

## A compromised innate immunity response hampers parasite elimination in hosts

Adaptive immunity presents a significant “memory” trait, which induces stronger and faster immune responses upon second exposure to pathogens, thus providing long-term protection against infection. Evidence suggests that robust memory responses develop after reinfection of viruses and bacteria, whereas the host’s immune system appears to have only partial resistance to parasitic infection (43–45). The complete protective effect mediated by immunity (referred to as sterilizing immunity) is only observed in cutaneous leishmaniasis caused by tropical *Leishmania* (46). In contrast, most hosts establish non-sterilizing immunity, which maintains a lower parasite burden in hosts and fails to provide adequate protection against the reinfection of parasites. This results from the well-known fact that parasites are equipped with superior immunoregulatory skills, thereby suppressing and converting the host immune response away from lethality and creating surroundings favorable for survival.

Due to differential parasite size, antigen composition, and life cycles, host immune responses triggered by different types of parasites present specificity to some extent yet share many similarities. In general, the protozoan and the early stage of helminth infection tend to trigger Type 1 immunity (47). However, with the progress of helminth infection, the immune response is biased toward Type 2 immunity (48). Inflammatory monocytes (Mo) and macrophages (Mφ) are important in killing invasive parasites *via* two mechanisms. Upon infection, Mo triggers the production of reactive oxygen species (ROS), provoked by respiratory burst during phagocytosis, and contributes to early parasite control.

In contrast, Mφ synthesizes nitric oxide (NO) through inducible nitric oxide synthase (iNOS) after activation by IFN-γ (47). Following initial interaction between parasites and pattern recognition receptors (PRRs) (*e.g.*, Toll-like receptors, TLRs; C-type lectin receptors), antigen-presenting cells (APCs), including Mo and DCs initiate and maintain Th1 immune responses through antigen presentation and production of inflammatory cytokines such as IL-12, TNF-α, IL-6. CD4^+^ T and CD8^+^ T cells and NK cells respond to IL-12 by secreting IFN-γ, which further instructs Mφ to activate the killing mechanism and enhance inflammatory cytokines production.

However, to resist hosts’ immune systems, parasites have evolved a series of immunomodulatory strategies to precisely suppress or downregulate immune responses, thereby escaping the elimination of immunity. The immunoregulatory events are initiated from the beginning post-infection. The outcome is an optimized environment featuring less aggressive immune cells, decreased pro-inflammatory cytokines (*e.g.*, IFN-γ, IL-1, IL-12, TNF-α), and increased inhibitory cytokines (*e.g.*, IL-10 and TGF-β). It is reported that *Leishmania* and *Toxoplasma gondii* can suppress the production of inflammatory cytokines, chemokines, and the expression of costimulatory molecules in Mφ *via* interference with MAPK, NF⁃κB, STAT3, or activation of STAT6-PPARγ/δ pathways, thus promoting Mφ to differentiate towards anti-inflammatory M2 phenotype. The activation of M2 Mφ is synchronized with the tilt of immune response to Th2, which is detrimental to the elimination of protozoa (49–59). Mφ and DCs are pivotal first responders of the innate immune system against early stage of malaria infection. Mφ controls the parasite burden in early infection stages primarily through phagocytic clearance. DCs are highly efficient in producing chemokines and cytokines in response to malaria infection, and interplay with other cells in the innate and adaptive immune systems. IL-12 produced by DCs activates NK cells, inducing secretion of IFN-γ, which facilitates Th1 and effector T cell responses (60). Massive production of IFN-γ contributes to the control of parasitemia by activating Mφ and neutrophils for enhanced phagocytic activity and consequently, parasite clearance (61, 62). However, Mφ becomes immunosuppressive following internalization of infected erythrocytes, hemozoin or merozoites (63–67), resulting in the inability to produce chemokines and cytokines, which is detrimental to the control of *Plasmodium* burden (68).

For helminths, the immunomodulatory strategies share many similarities with protozoa yet present quite diversities (69, 70). As the infection progresses, helminths induce the proliferation of Tregs and immunoregulatory Mo along with high levels of immunosuppressive cytokines TGF-β and IL-10 (48). Numerous studies have shown that helminths facilitate the activation and proliferation of Th2 cells and M2 Mφ. These cell types are involved in the anti-inflammatory responses to infection or tissue damage (71). *Heligmosomoides polygyrus bakeri* (*Hpb*) and its products are found to affect the metabolism and effector functions of eosinophils, group 2 innate lymphoid cells (ILC2), Mφs and neutrophils, which play important roles in the early type 2 immune response against helminth infection (72–74). Mice infected with *Strongyloides venezuelensis* (*Sv*) are protected from *Nippostrongylus brasiliensis* (*Nb*) infection for at least 3 months, and this protective effect may be related to the induction of long-term trained immunity programs in eosinophils and ILC2 (75, 76). House dust mite (HDM)-induced trained eosinophils provide protection against subsequent *Ascaris* infections in mice, and this protective effect against helminth infections is mainly dependent on Th2 cells (77–79). Thus, helminths and their products play key roles in the induction of trained type 2 immunity. It is found that Mφ trained with *Fasciola hepatica* total extract (FHTE) exhibit enhanced anti-inflammatory trained immunity that is mediated by histone methylation, suppressing effector Th1 and Th17 responses (80). In addition, Mφ from mice treated with FHTE express alternative activated macrophage (AAM) markers and produce large amounts of IL-10 and IL-1RA in response to FHTE or TLR ligands and inhibit the production of TNF and IL-12p40. Thus, by exposure to helminth products, innate immune cells can be trained *in vivo* or *in vitro* to be more anti-inflammatory, thereby protecting mice from the induction of T cell-mediated autoimmune diseases (80).

Taken together, parasites apply several tactics to subvert the early fatal Th1 response, inducing an environment characterized by a state of immunosuppression or a moderate Th2-biased immune response in hosts. Despite Th2-type immune response matters in protective immunity against helminth infection, it rarely kills parasites, only limiting their infection and weakening their survival and fertility. As a result, parasitic infection tends to cause long-term and asymptomatic infection in hosts, while the failure of elimination brings about chronic damage to hosts’ health and finally accumulates horrible consequences. Moreover, as the immune system cannot successfully control the parasitic infection in the early stage, parasites stand a chance to multiply rapidly in hosts, and the afterward inappropriate-activated systemic inflammation ultimately leads to immunopathological changes. In addition, suppression of the immune system also impairs vaccine efficacy to some extent, which undoubtedly intensifies the difficulty of preventing and controlling parasitic diseases (81–83). Consequently, a universal way to combat most types of parasites may be to reverse the suppressed state of the immune system and increase early Th1 or innate immunity to kill newly invading larvae, minimize the parasitic burden or even achieve complete prevention.

**Figure 1:**
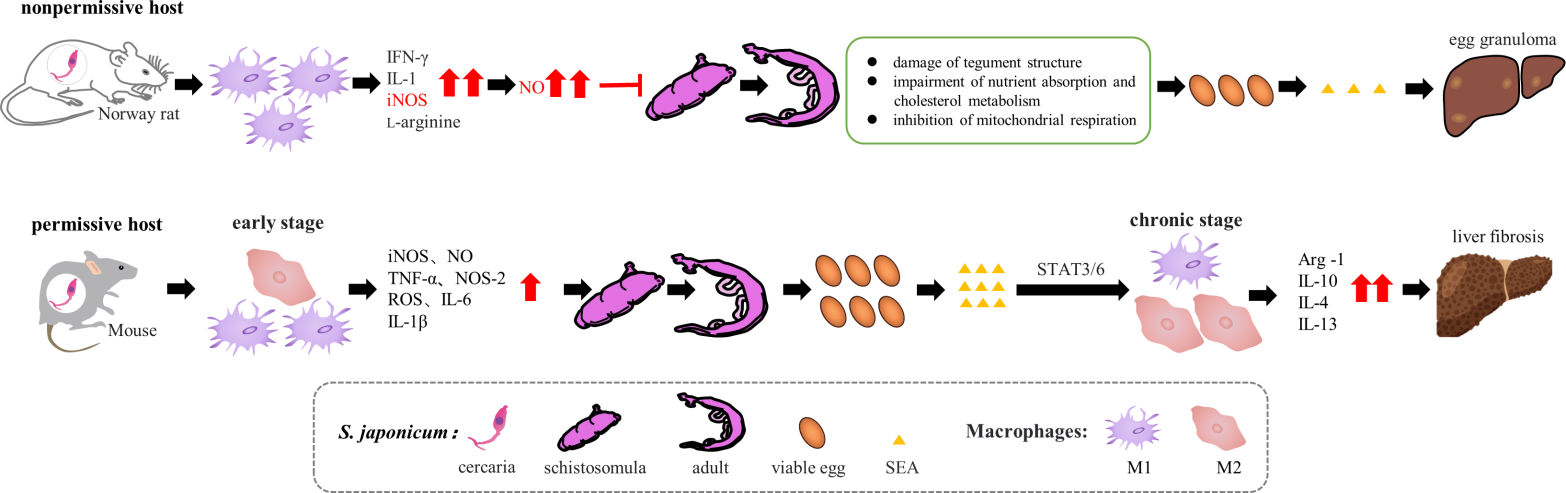
The immune profiles of *S. japonicum* infection in permissive and non-permissive hosts. In the Norway rats (non-permissive host), immune cells, mainly M1 polarized Mφ, produce nitric oxide (NO) through L-arginine metabolism by iNOS in response to cytokines (*e.g.*, IFN-γ, IL-1). NO leads to impaired nutrient uptake and cholesterol metabolism in the parasite and the disruption of mitochondrial function and inhibition of mitochondrial respiration. These responses restrain the parasite’s viability in hosts, reducing the number of viable eggs and their produced soluble egg antigen (SEA), consequently leading to decreased liver fibrosis. In the permissive hosts (*e. g* C57BL/6J mice), the early stage of *S. japonicum* infection induces M1 Mφ polarization, leading to the secretion of inflammatory factors. However, In the chronic phase of infection, the SEA can induce M2 Mφ polarization through STAT3/6 pathways, followed by the secretion of anti-inflammatory factors, which promotes the progression of liver fibrosis.

Interestingly, an analysis of the non-permissive host of *Schistosoma japonicum*-*Microtus fortis* is obtained from the comprehensive data of a cytokine chip assay, transcriptome, and metabolome (84), which may dramatically support our opinion. *M. fortis* presents natural resistance against *S. japonicum*, and the invading cercaria ultimately dies in the livers of *M. fortis*. After 12 days of infection, several white nodules composed of dead cercaria and a large number of surrounding inflammatory cells are observed on the surface of *M. fortis* liver and disappear for the next two weeks. In addition, no eggs, adult worms, or schistosomula are found in *M. fortis*. Furthermore, cytokines such as IL-1β, IL-2, IL-12, IFN-γ, GM-CSF, and MCP-1 are significantly higher than those in C57BL/6J mice (permissive host for *S. japonicum*) (**Figure 1**). Further analysis uncovers that differentially expressed metabolites, including unsaturated fatty acids, quasi-vitamins, and amino acids, are detected in the metabolomic pathway analyses, which relates to the strengthened function and activity of innate immune cells. These results indicate that early initiation of a solid anti-infectious immune response contributes to the natural resistance of *M. fortis* against *S. japonicum* infection. Further studies revealed that iNOS is highly expressed in Norway rats (85) (**Figure 1**), and iNOS interferes with mitochondrial respiration and energy production by producing NO (86, 87), thereby inhibiting *S. japonicum* growth (88), formation, and development of female and male reproductive organs, and oviposition (89), leading to the production of unfertilized eggs (90). The above effects significantly reduce the viability of *S. japonicum* (88, 91) and jointly disrupt the formation of granulomas induced by *S. japonicum* eggs (88). Moreover, NO can impair the tegument of *S. japonicum* (90), with adverse effects on its nutrient absorption and cholesterol metabolism (92, 93). Thus, enhancing the initial innate immune response may be essential to designing novel parasitic vaccines.

## Trained immunity boosts innate immune response *via* metabolic and epigenetic reprogramming

Previous opinions hold that only the adaptive system presents the “memory” trait when exposed to the reinfection of pathogens. Instead, trained immunity (TI), also termed innate immune memory, represents an adaptive form of innate host defense mechanisms or de facto innate immune memory. Following exposure to a specific infectious agent or vaccine, innate immune cells (mainly Mo/Mφ, NK cells, and innate lymphoid cells) can respond more rapidly and more strongly to secondary attacks by homologous or even heterologous pathogens (42, 94, 95). However, trained immunity can lead to aberrant inflammatory responses in cases such as autoimmunity (96). The mechanism of trained immunity involves long-term metabolic and epigenetic reprogramming of cells associated with strong immune responses (42, 97, 98).

**Figure 2:**
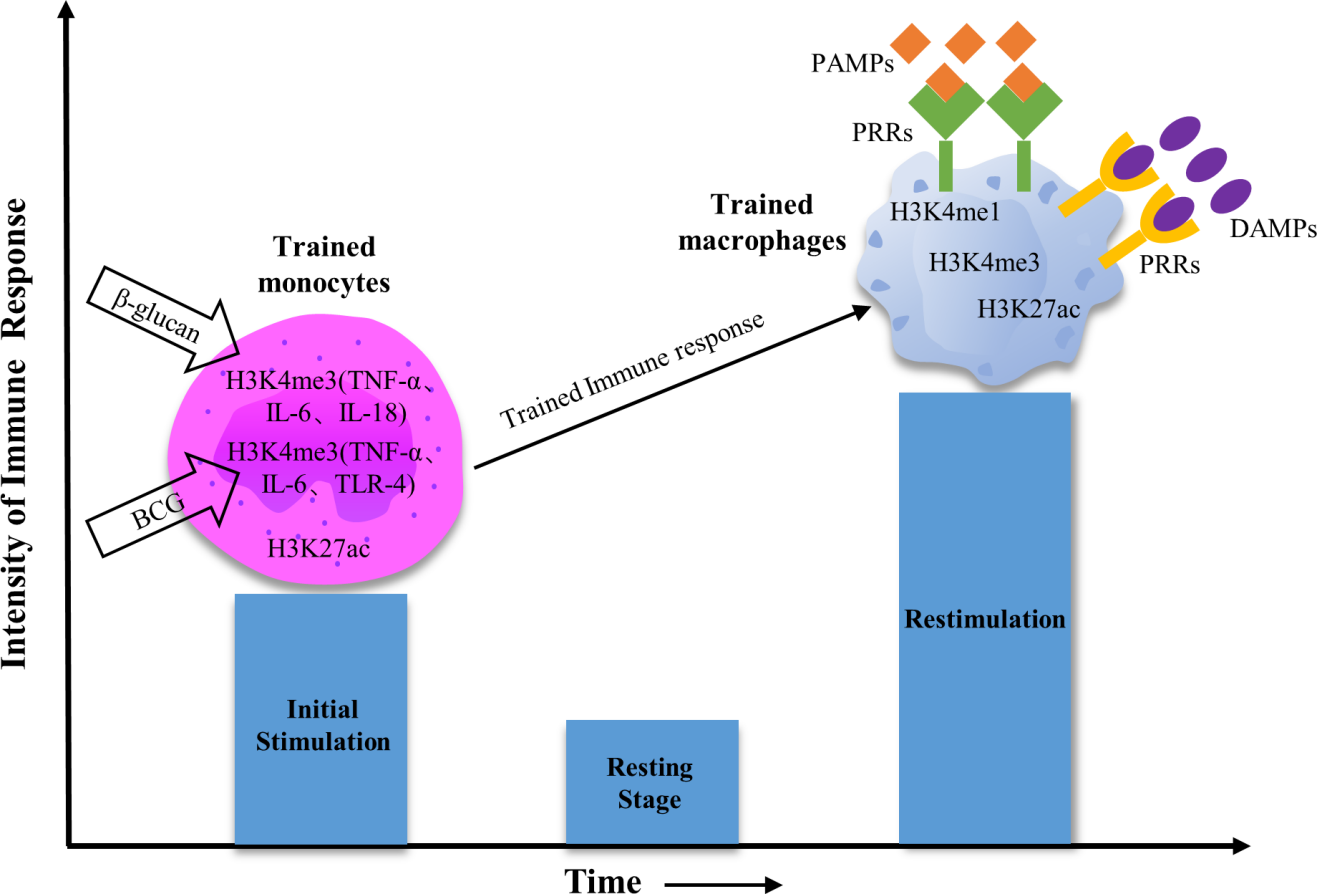
Epigenetic modifications in trained monocytes/macrophages. Initial stimulation of monocytes with β-glucan or BCG leads to histone modifications, which leave epigenetic reprogramming marks on activated genes. Among them, the increased H3K4me3 levels in the trained monocytes were mainly reflected in the promoter regions of genes associated with the expression of pro-inflammatory cytokines. Because chromatin markers are partially removed after primary stimulation, these genes are partially repressed during the resting period and cannot be transcribed. After re-stimulation with PAMPs or DAMPs, trained macrophages can undergo rapid gene transcription machinery and increase the acetylation, monomethylation, and trimethylation of gene promoter regions.

**Figure 3:**
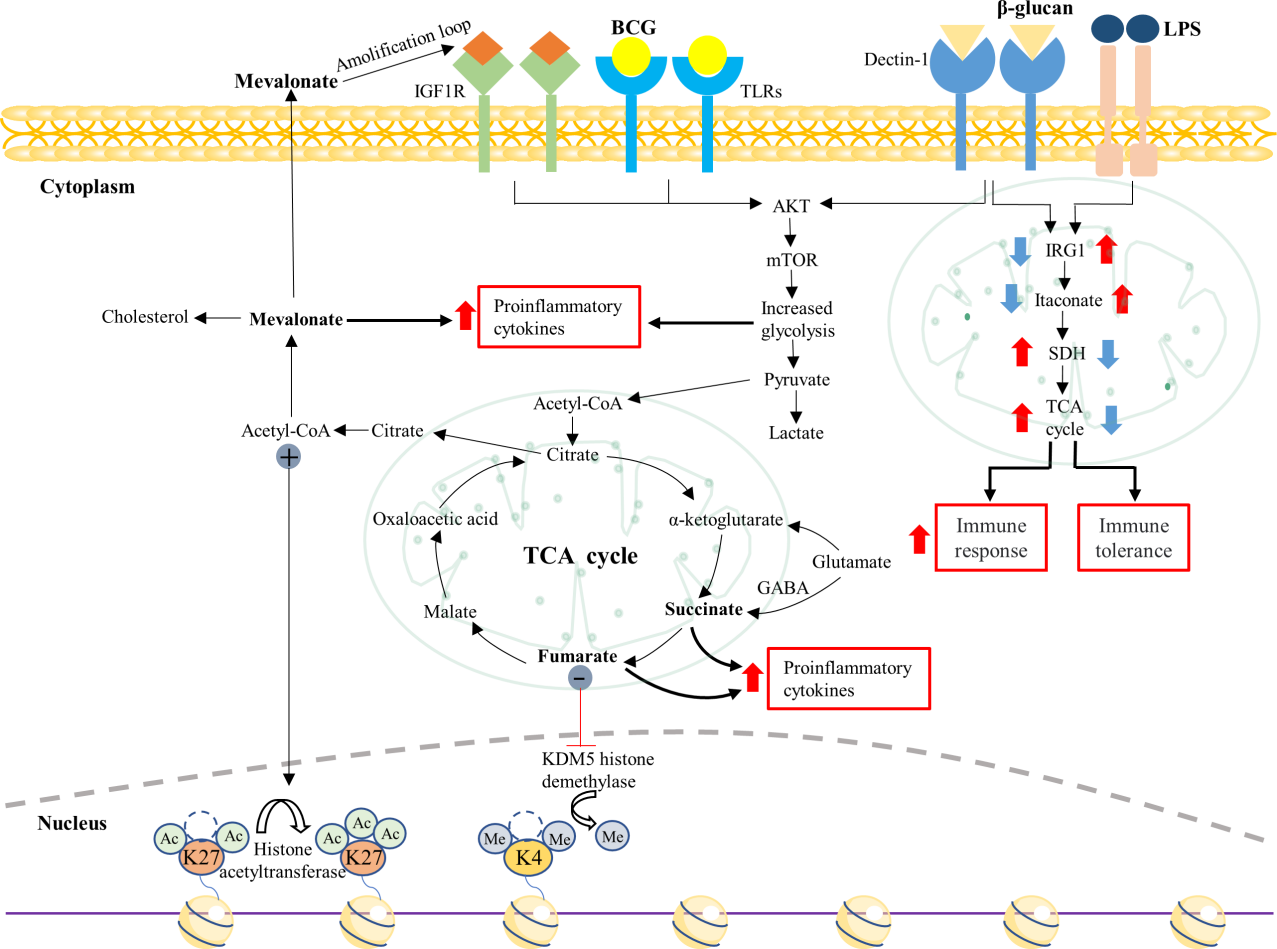
Interaction of metabolic and epigenetic events in trained monocytes/macrophages. Trained immunity is induced in Mo/Mφ by specific ligands (*e.g.*, β-glucan and BCG) *via* binding to PRRs (*e.g.*, Dectin-1 and TLRs), triggering a series of intracellular cascades. In the process, accumulated intermediate metabolites can regulate the innate immune response via complex mechanisms. For example, fumarate and acetyl-CoA can inhibit KDM5 histone demethylase and activate histone acetyltransferase, leading to specific changes in histone methylation and acetylation in the trained cells. The metabolite mevalonate can induce metabolic and epigenetic reprogramming in the monocytes/macrophages *via* interacting with IGF1R. In addition, β-glucan can regulate the IRG1-itaconate-SDH axis to enhance the pro-inflammatory response in trained monocytes/macrophages. PRR, pattern recognition receptor; TCA, tricarboxylic acid; IGF1R, insulin-like growth factor 1 receptor; IRG1, immune-responsive gene 1; SDH, succinate dehydrogenase.

In contrast with adaptive memory, which lies on the gene recombination of antigen receptors and proliferation of specific lymphocyte clones, TI is triggered by the interplay between pathogen-associated molecular patterns (PAMPs) or damage-associated molecular patterns (DAMPs) and pattern recognition receptors (PRRs) on the innate immune cells, followed by long-term metabolic and epigenetic changes (99, 100) (**Figures 2**, **3**). TI can be initiated immediately after infection but usually lasts only weeks to months after stimulation. The bacterial or fungal ligands such as BCG and β-glucan have been identified as the inducers of TI (**Figures 2**, **3**). However, on the opposite of the training event, a high amount of lipopolysaccharide (LPS) can induce epigenetic silencing and a weakened innate immune response toward the second challenge, which is called tolerance (100) (**Figure 3**).

The phenomenon of TI has been reported in Mo/Mφ, NK cells, and innate lymphoid cells (99, 101–104). Notably, the underlying mechanisms of TI in Mo/Mφ have been extensively investigated. Mo and Mφ belong to the mononuclear phagocyte system. Mo is an intermediate developmental stage between bone marrow precursor cells and tissue Mφ (105). Circulating Mo has critical biological functions, including maintaining homeostasis of the organism through patrol and repair functions (106) and generating inflammatory responses during infections in the organism (107). During infections, mature blood inflammatory Mo migrates to inflamed tissues and differentiates into Mo-derived Mφ, clearing pathogens and restoring tissue integrity (108). In an *in vitro* study, Mo was cultured in a human serum medium to induce their differentiation into Mφ (109). In the process, Mo that underwent transient priming by β-glucan differentiated into trained Mφ (101). It is reported that β-glucan induces an epigenetic modification involving many promoters and distal elements and the enhancers on differentiation-sensitive promoters (102). Thus, trained Mo and differentiated Mφ produce higher inflammatory factors in response to stimuli such as ligands targeting pattern recognition receptors (*e.g.*, LPS and Pam3CSK4). This is beneficial for resistance to reinfection by the same or different pathogens, including parasites (110). Here, we reviewed how metabolic and epigenetic events determine TI in Mo/Mφ.

## Metabolic reprogramming

### (i) Glycolysis

In the resting state, the energy supply in Mo/Mφ relies on oxidative phosphorylation (OXPHOS); however, these cells prefer aerobic glycolysis upon activation (111, 112). The metabolic shift can provide Mo/Mφ with essential precursors for synthesizing nucleotides, amino acids, and lipids and is considered one of the prerequisites for TI (113). It is reported that the metabolic phenotype of β-glucan-trained Mo is characterized by increased glucose consumption, lactate production, the glycolytic pathway, pyruvate conversion, and decreased OXPHOS (114). β-glucan can act on Mo *via* DC-associated C-type lectin-1 (Dectin-1) receptor to activate the AKT-PI3K pathway, subsequently activating mTOR/HIF-1α (115). Moreover, several studies have demonstrated that the metabolic transition from OXPHOS to aerobic glycolysis mediated by Akt-mTOR-HIF-1α is the metabolic basis of TI (102, 116, 117) (**Figure 3**). In addition, BCG-induced TI also involves the genetic variation of glycolytic rate-limiting enzymes (*e.g.*, hexokinase 2) in Mo and alteration of AKT/mTOR/HIF-1α pathway (118).

### (ii) TCA cycle

Several intermediates produced in the TCA cycle can regulate the TI in Mo/Mφ (**Figure 3**). Citric acid is mainly converted from pyruvate and α-ketoglutarate, the metabolite of glutamine (119, 120). The citric acid in the cytosol can be converted to acetyl coenzyme A (AcCoA), which acts as an acetylation carrier to promote massive acetylation of histones (*e.g.*, H3K27ac) (**Figure 3**) and is closely associated with the intrinsic epigenetics of trained immune cells (115, 121). Succinate and fumarate are significantly increased in trained Mo/Mφ (122) (**Figure 3**). On the one hand, large amounts of succinate and fumarate inhibit the degradation of HIF-1α by prolyl hydroxylase (PHD), thus maintaining the stability of HIF-1α. Then, HIF-1α can induce lactate production, disrupt TCA cycling, and upregulate IL-1 expression (123, 124). On the other hand, succinate and fumaric acid can act as antagonists of histone and DNA demethylases to promote the methylation of histones and DNA (102, 125), which leads to chromatin de-folding and promotes transcription and expression of pro-inflammatory factors (110).

Furthermore, the stimulation of LPS to Mo can induce increased expression of IRG1, which causes massive synthesis of itaconate (126), inhibiting the expression of SDH (127, 128). Itaconate can induce immune tolerance by inhibiting cytokine production (129). Inhibition of SDH expression causes an impeded conversion of succinate to fumarate, inhibiting the TCA cycle’s activity (127, 128) and leading to the development of immune tolerance. β-glucan-induced Mo can inhibit LPS-induced IRG1 expression, thereby blocking the detrimental effects of itaconate on immune tolerance (126) and increasing the expression of H3K27ac at the SDH level, restoring cellular metabolism (130), restoring the production of pro-inflammatory cytokines (131), and thereby reversing LPS-induced immune tolerance (**Figure 3**).

### (iii) Glutamine metabolism

Glutamine (Gln) metabolism of β-glucan-trained or BCG-trained Mφ increases in some research (118) (**Figure 3**). Glutamate and α-KG that are catabolic products of Gln, as well as succinate generated by the gamma-aminobu-tyricacid (GABA) pathway, complement the intermediates in the TCA cycle (115, 132), which promotes TI. Furthermore, Gln catabolism products themselves can regulate TI through several pathways. Firstly, AcCoA produced from glutamate is a substrate for histone acetyltransferase, which promotes the acetylation of H3K27 (H3K27Ac) in histones in Mo (102, 133), in turn inducing TI. Secondly, glutamine supplementation of the TCA cycle generates large amounts of malate, which can be transported to the cytosol for pyruvate production. The massive synthesis of pyruvate and the activation of the mTOR-HIF-1α pathway will further promote the aerobic glycolytic process, facilitating the TI process (134, 135).

### (iv) Cholesterol synthesis pathway

In the culture system of β-glucan-trained Mo/Mφ, those trained cells show enhanced glycolysis and disrupted TCA, which allows a large amount of pyruvate to be converted to AcCoA. Then, AcCoA can directly synthesize fatty acids or cholesterol via the mevalonate (MVA) pathway (136–140). In line with this, over half of the trained cells exhibit upregulation of genes associated with cholesterol synthesis-related pathways (115). Mevalonate (MVA), a key intermediate in this pathway, also accumulates in the Mo/Mφ. Notably, MVA is recently reported to activate the mTOR pathway by interacting with the Insulin-like growth factors-1-receptor (IGF1R) to induce TI in Mo/Mφ (141) (**Figure 3**).

## Epigenetic reprogramming

Mo and Mφ express PRRs, which makes them able to accept and respond to PAMPs and DAMPs stimulation (142, 143). In this process, these cells undergo epigenomic programmed changes, referred to as epigenetic reprogramming, representing one important TI induction mechanism (**Figure 2**). For example, Mφ trained with β-glucan or BCG is often accompanied by changes in H3K4me3 at the promoters of genes encoding inflammatory cytokines and extensive changes in other chromatin markers (H3K4me1, H2K27ac, and H3K9me2, among others) (102, 114). These changes persist for weeks to months and determine the extent or type of immune response of the cells upon reinfection (144, 145). It is reported that Histone acetylation marks (*e.g.*, H3K27ac) are short-lived and disappear upon removal of the stimulant, while methylation marks are long-lasting (146–149). H3K4me1, as an epigenetic marker, is actively present in enhancers, which maintains the long-term immune memory characteristics and enables Mo/Mφ cells to produce faster and more robust responses upon re-stimulation (148–150).

Furthermore, the immune tolerance process is accompanied by altered histone modification levels. In tolerance experiments with high doses of LPS-induced sepsis, changes in histone H3K4me3, H3K27me2, and histone methyltransferase complexes (102, 151, 152) can downregulate the genes encoding inflammatory factors while upregulating the anti-inflammatory factor genes (153, 154). On the contrary, the ultra-low doses of LPS induce a trained response and enhance the inflammatory response upon LPS re-stimulation (155, 156). In a few pretreatment experiments, low doses of LPS can protect against pathogen infections via epigenetic modifications to increase the expression of inflammatory factor genes, thereby enhancing the phagocytic and killing activity of Mφ (157).

## The interaction between metabolic and epigenetic reprogramming

Mo and Mφ stimulated with microorganisms, or endogenous ligands, produce numerous metabolites, which can regulate the function of enzymes responsible for modifying histones and DNA, thereby altering the intrinsic epigenetic landscape. For example, α-KG acts as a cofactor for the activation of the histone demethylase JMJ family (Jumonji) to promote histone demethylation (102, 125); AcCoA provides acetyl substrate for histone acetyltransferases (158); succinate inhibits histone demethylation and inhibition of succinate dehydrogenase (SDH) which inhibits histone methylation (159). Moreover, histone lysine-specific demethylase 1 (LSD1) of the JmjC and JmjD families require α-KG as a cofactor for the demethylation process (156), whereas succinate and fumarate inhibit its demethylation process (160). It is reported that high concentrations of fumarate and succinate can enhance the 3-methylation of histones H3K4 and H3K27 by inhibiting KDM5 histone demethylase (responsible for H3K4 demethylation) and JMJD3 histone demethylase, respectively, which in turn promotes the expression of pro-inflammatory cytokine genes (99, 115, 125, 161, 162). Thus, metabolic and epigenetic reprogramming are tightly integrated and sequentially regulated to train the immune process.

## Trained immunity provides a novel perspective for designing anti-parasitic vaccines

When it comes to advancing trained immunity in parasitic vaccines’ development, investigations are still in their infancy, yet with a steady-climbing trend. It is reported that stimulation of human adherent PBMCs with *Plasmodium falciparum*-infected red blood cells or the malaria crystal hemozoin followed by stimulation with TLR2 ligands can induce increased production of pro-inflammatory cytokines (163). This finding proposes the induction of trained immunity as a cutting edge in parasitic infection. *Bacillus-Calmette Guérin* (BCG) is a well-known inducer of trained immunity, which can induce long-lasting metabolic and epigenetic changes in Mo/Mφ or NK cells and, consequently, the non-specific memory features (102, 114). The demographic epidemiological survey has indicated that BCG vaccination reduces malaria-specific mortality in malaria-endemic areas (164). To support this hypothesis, BCG-mediated trained immunity boosts the pro-inflammatory cytokine responses (99, 165), thereby resisting the infection towards other pathogens (99, 165).

Moreover, BCG training can lead to early expression of CD69 in NK cells and HLA-DR expression in Mo, which can help reduce parasitemia (166). Furthermore, a recent study has shown that BCG-trained human Mo can enhance the killing of *L. braziliensis*, *L. infantum*, and *L. amazonensis* by increasing the production of reactive oxygen species (167). Moreover, BCG vaccination can reduce parasite load and lesion size in organisms infected with *L. braziliensis* and reduce the risk of transmission of *L. amazonensis* to other organs in infected mice (167). Furthermore, β-glucan can also induce trained immunity to increase resistance to *Leishmania* spp. It is reported that β-glucan-trained Mo, by interacting with dectin-1 and the complement receptor 3 (CR3) receptors (101), can increase cytokine production, especially IL-6, thereby improving the host’s ability of phagocytosis and killing of *L. braziliensis* (168). β-glucan training affects gene regulatory mechanisms, causing altered histone methylation (namely H3K4me1 enrichment), which leads to transcriptional induction of IL-32 by Mφ to produce higher levels of antimicrobial peptides such as cathelicidin and β-defensin 2 (168). These antimicrobial peptides can help Mφ kill and eliminate *Leishmania* spp (169, 170). In addition, β-glucan can stimulate a long-term pro-inflammatory Mφ phenotype (168), thereby enhancing the response to parasite infection, which is beneficial for controlling the parasite burden.

Given the potential role and promise of trained immunity in the control of parasitic infections, integration of the innate immune response in the form of trained immunity into the design of future vaccines could improve vaccine efficacy. It could be thus hypothesized that a vaccine targeting at the same time innate and adaptive immune memory would be more successful than existing vaccines against infections, including parasitic infections, that only aim to boost adaptive immune responses (155). It was found that rBCG strains first induced TI against Babesia in the early stages of parasitic infection and then specific antigens, thereby inducing acquired immune memory in the later stages of parasitic infection. The ability of BCG to deliver rhoptry-associated antigen 1 (RAP-1) of *Babesia bovis* was further confirmed in mice, which supported the hypothesis that rBCG could be used as a component of an anti-Babesia vaccine (171). Therefore, BCG-induced TI and rBCG may be novel strategies to induce protection against acute babesiosis in humans and cattle. This approach can be used either alone to control acute babesiosis infection and prevent deaths or with specific anti-babesiosis vaccines to prevent persistent babesiosis infection. The strategies described above have great potential to reduce the parasite load in the vertebrate host and reduce the risk of parasite transmission, thus favoring the control of disease progression. In addition, BCG-induced TI can play a considerable role in eradication protocols for babesiosis (172).

These studies demonstrate the powerful effect of trained immunity in defending against parasitic infections. It is most likely that boosting innate immunity *via* trained immunity may also contribute to eliminating a broader spectrum of parasites, although the main findings are only observed in *Plasmodium*, *Leishmania*, and *Babesia*-infected models.

## Rewiring of metabolism and epigenetics of innate immune cells: a cutting edge approach for the development of novel parasitic vaccines

Considering the problems of parasitic vaccine development, it is very urgent to design more effective parasitic vaccines in view of novel insights. Epidemiological studies have indicated that, in addition to specific disease-protective effects, vaccines against infectious diseases have beneficial or detrimental non-specific effects on other pathogens (173–175). Intriguingly, the emerging trained immunity may provide a possible explanation for non-specific effects. The concept of “trained immunity-based vaccines (TIbV),” proposed by Sánchez-Ramón et al. in 2018, contributes to the exploitation of parasitic vaccines to expand their therapeutic efficacy (176). TIbV challenges traditional vaccines in several ways (176), although its clinical utility and immunological implications warrant further study. For example, TIbV may not be limited to the formulation of its antigen and is based on trained innate immune cells to provide non-specific protection against different pathogens. Furthermore, TIbV has self-adjuvant characteristics that enhance adaptive immune responses to autoantigens and bystander antigens. TIbV may open a new way to develop broad-spectrum parasitic vaccines in this context.

**Figure 4:**
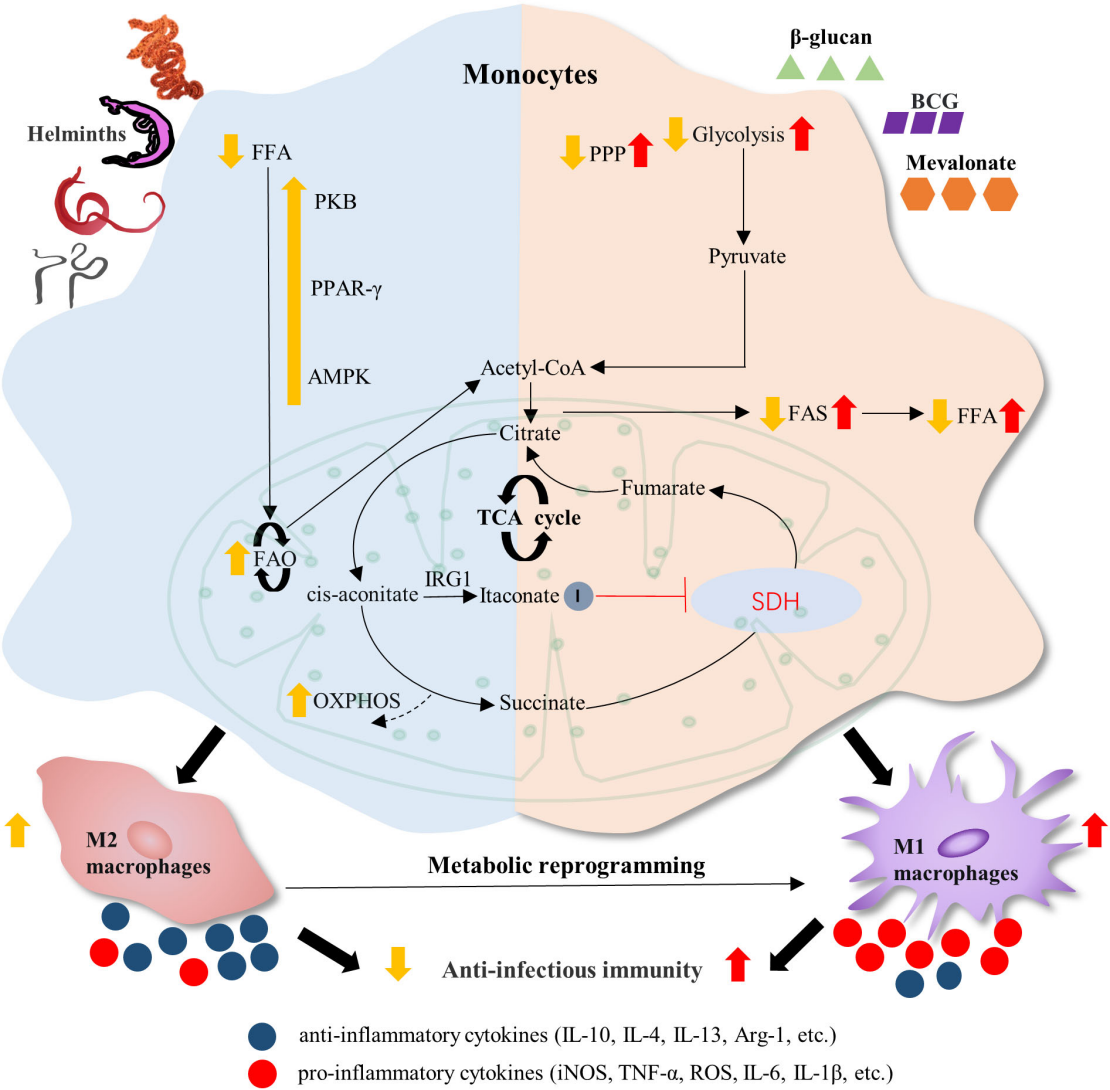
Proposed mechanisms of trained immunity in elevating anti-parasitic innate immunity. Helminth infection can induce the M2 polarization of macrophages in the chronic stage, producing a series of anti-inflammatory cytokines and weakening anti-infectious immunity. Stimulants such as β-glucan, BCG, and mevalonate may train the monocytes and alter the metabolic flux, which induces the M1 polarization of macrophages and enhance the anti-parasitic immunity. FFA, free fatty acid; PKB, protein kinase B; PPAR-γ, peroxisome proliferator–activated receptor-γ; AMPK, adenosine monophosphate (AMP)-activated protein kinase; FAO, fatty acid oxidation; PPP, pentose phosphate pathway; FAS, fatty acid synthesis; IRG1, immune-responsive gene 1; SDH, succinate dehydrogenase; OXPHOS, oxidative phosphorylation.

To develop effective vaccines by enhancing the anti-parasite immunity of the host, it is crucial to gain a deeper understanding of parasitic infection-driven immunomodulatory mechanisms. TI has been reported in a variety of cell populations, including Mo/Mφ, NK cells, innate lymphoid cells (ILCs) and polymorphonuclear leukocytes (77, 101, 116, 157, 177–182). This review focuses on the design of novel parasite vaccines based on Mo/Mφ, as the two types of cells are currently more comprehensively investigated in the field of parasitic infections and TI induction. Mφ can be polarized into classical activated Mφ (M1 Mφ) and alternative activated Mφ (M2 Mφ) under the stimulation of different environments (183) (**Figure 4**). In general, M1 Mφ is related to the initiation and maintenance of the inflammatory process and eliminates parasites by producing TNF⁃α, IL⁃12, and iNOS (184). However, M2 Mφ can metabolize L⁃arginine into proline and polyamine through Arg1 to promote the survival of parasites in the host, which is related to the regression of the inflammatory process and tissue repair (185). Thus, M1 Mφ is conducive to controlling parasite infection (184), and M2 Mφ is beneficial for parasite survival in the host (185). However, how to reverse Mφ M2 to M1 is a shortcoming in the field. Interestingly, the emerging discipline of TI may enhance the anti-infectious immunity of Mo/Mφ *via* epigenetic and metabolic events (186, 187) (**Figure 3**). Thus, rewiring metabolism and epigenetics in these innate cells may be the principle for designing novel parasitic vaccines.

## Induction of M1 Mφ

Mφ is an essential component of the innate immune system and the first line of defense against parasitic infections. Metabolic reprogramming is the critical event in the phenotypic differentiation of Mφ. The M1 type Mφ is associated with an inflammatory response with a predominantly glycolytic response and a disruption of TCA following citric and succinic acid that leads OXPHOS to be impaired. Glycolysis can supply M1 cells with energy to produce more pro-inflammatory factors to rapidly kill microorganisms and adapt to the hypoxic microenvironment of tissues (108, 188, 189). Activation of the pentose phosphate pathway (PPP) is beneficial to M1 cells to maintain inflammatory response (190) (**Figure 4**). However, M2 Mφ predominates in the chronic or advanced stages of helminth infection (**Figure 4**). It is reported that helminth infection promotes IL-4 production and maintains the OXPHOS of Mφ to differentiate into M2 Mφ (191–193). OXPHOS continuously provides a large amount of energy required for tissue remodeling and repair (108, 194, 195). Furthermore, both adults and eggs of *Schistosoma mansoni* can induce M2-type polarization of Mφ by peroxisome proliferator-activated receptor γ (PPARγ) activation, which is beneficial to parasite survival (70) (**Figure 4**). Thus, M1 Mφ plays a crucial role in anti-infectious immunity through metabolic reprogramming.

Over the past few years, discoveries in immunology have modified the current paradigm. It turns out that the innate immunity associated with hyper-responsiveness often leads to enhanced host defense against secondary infections, which is termed trained immunity (TI) (196). Immunomodulatory compounds, including β-glucan and BCG, are good inducers of TI *via* reprogramming the metabolic and epigenetic landscape in Mo and Mφ to augment the host response to parasitic infection (102, 114, 168, 197, 198). Mo trained by β-glucan can induce TI, causing increased H3K27ac modification and deposition of H3K4me1 and H3K4me3 gene promoters, leading to transcriptionally active chromatin (102, 197); it also can cause the increased expression of enhancer proximal genes, favoring the generation of a more robust transcriptional response (197). Moreover, β-glucan is reported to induce metabolic changes *via* the mTOR/HIF-1α pathway (114), resulting in a metabolic shift from OXPHOS to aerobic glycolysis (197), which facilitates the induction of Mo polarization into M1 Mφ. Trained Mφ will have elevated succinate levels in the TCA cycle, which can cause enhanced H3K27me3 in the M2 Mφ gene by inhibiting JMJD3 activity, leading to suppression of M2 Mφ expression (102, 199).

Furthermore, BCG recognizes different *Mycobacterium tuberculosis* molecular patterns through primed intracellular Toll-like receptors (TLRs) 7 and 9 in Mφ, activating intrinsic immune transcription and gene expression profiles through the NF⁃κB pathway to TLR signaling (200). BCG-trained Mφ cause increased expression of H3K4me3 and H3K27ac at the promoter sites of genes essential for glycolysis (114), as well as decreased expression of H3K9me3 (161), resulting in changes in cellular metabolism (118). Moreover, it is reported that the increased glycolysis and oxygen consumption (118) is induced through the Akt/mTOR pathway (114). Upregulation of glutamine metabolism and accumulation of succinate are involved in IL-1β production via upregulation of HIF-1α (124). Similarly, metabolites such as ferredoxic acid and 2-hydroxyglutaric acid can affect DNA demethylation enzymes (201), thus favoring chromatin condensation and preventing transcription factor binding and gene silencing (202, 203). The TI triggered by BCG leads to a sustained enrichment of H3K4me3, favoring the production of pro-inflammatory cytokines and enhancement of gene promoter regions associated with the expression of intracellular signaling molecules in Mφ (190). All these pathways induce Mφ polarization toward M1 Mφ. Thus, β-glucan and BCG can induce Mφ polarization toward M1 Mφ through the interplay and interaction between epigenetic reprogramming and cellular metabolic reprogramming to enhance anti-infectious immunity.

In addition, Mo and Mφ remove pathogens as part of innate immunity and contribute to adaptive immunity as antigen presentation cells. Upon pathogen invasion, PRRs (*e.g.*, TLRs) expressed on the inflammatory Mφ initiate the innate immune signaling pathways and trigger a variety of pro-inflammatory cytokines production, which in turn instructs a pro-inflammatory adaptive immune response involving primarily Th1 cells (204, 205). During a Th1 response, T cells, specifically CD4^+^ Th1 cells, could play an integral role in suppressing parasite growth by producing IFN-γ and IL-2 (206–208). Therefore, inducing M1 Mφ may aid in optimizing vaccine strategy to elicit broadly protective innate immunity and parasite-specific adaptive immunity. In conclusion, future work on developing parasitic vaccines should further explore the pathways associated with the induction of Mφ polarization by parasite infection and focus on applying immune adjuvants such as BCG and β-glucan to alter the associated cellular metabolic and epigenetic reprogramming. Moreover, Mφ that undergoes metabolic reprogramming causes the accumulation of intermediate metabolites (124, 209–213). There is accumulating evidence showing that several metabolites are conducive to anti-infectious immunity due to their immunomodulatory properties (114, 115, 141), which may provide the development of vaccines with potent adjuvants.

## Reversal of immune tolerance

Parasitic infections in chronic stages often compromise the host’s anti-parasite immunity, termed immune tolerance. For example, high levels of iNOS and NF⁃κB expression were shown in the peripheral blood of primary patients with *Echinococcus granulosus*, while their low expression occurred in the relapsed patients (214). Thus, reversing the immune tolerance can boost anti-infectious immunity. Interestingly, β-glucan binding to dectin-1 is reported to initiate the phagocytosis, produce the superoxide *via* NADPH oxidase (215–217), and convert Mφ to the M1 phenotype *via* NF–κB–autophagy-dependent pathway (218). Moreover, in a model of immune tolerance induced by LPS in volunteers, β-glucan can partially reverse LPS-induced immune tolerance by modulating the cellular epigenome, which allows elevated expression of H3K27ac, thereby restoring the ability of Mφ to produce pro-inflammatory cytokines (197). In addition, it is reported that in a human sepsis model, β-glucan inhibits the expression of immune response gene 1 (IRG1), thereby downregulating the synthesis of itaconate and increasing the expression of succinate dehydrogenase (SDH) (**Figure 3**). This metabolic alteration can recover the ability of Mo/Mφ to produce high levels of pro-inflammatory cytokines responsible for secondary stimulation (110, 126, 197), thereby enhancing the killing of pathogenic organisms. Taken together, β-glucan can prevent or reverse immune tolerance by altering the relevant metabolic and epigenetic pathways and TI induction. It is speculative that such an effect of β-glucan may be a breakthrough point to ameliorate the parasite-induced immune tolerance in the host.

## Development of novel adjuvants

As a key vaccine component, the adjuvants can enhance the immune response through various activities (219, 220). They can bind to antigens and facilitate more accessible and efficient recognition of “non-self substances” by the body, thus triggering both innate and adaptive immune responses (221). Moreover, the adjuvants transport antigens to lymph nodes, thus facilitating T-cell antigen recognition. In addition, the adjuvants prolong the retention time of antigens, induce the release of pro-inflammatory factors, and increase the intensity of the local response at the injected site (222). Therefore, supplementation of adjuvants is necessary to develop a vaccine with high efficiency.

In the studies of parasitic vaccines, different types of adjuvants can significantly enhance the effectiveness of the vaccine. Monophosphoryl lipid A (MPL) is the component with adjuvant activity obtained after removing the toxic effects of bacterial lipopolysaccharides (223, 224). MPL^®^ is formulated in a variety of adjuvant platforms, including AS01 (MPL^®^ and QS-21 in phosphatidylcholine liposomes), AS02 (MPL^®^ and saponin QS-21 in an oil-in-water emulsion containing squalene and α-tocopherol) and AS04 (MPL^®^ adsorbed to aluminum oxyhydroxide). Numerous clinical studies have shown that TLR4 agonists such as AS01, AS04, and oil-in-water emulsion (GLA-SE) show excellent performance in the development of various vaccines against parasites (13, 225–233). In fact, the TLR4 ligand lipopolysaccharide is reported to induce epigenetic reprogramming of haematopoietic stem cells, leading to persistent changes in the accessibility of their myeloid lineage enhancers (234). Moreover, the TLR9 agonist CpG confers broad resistance to infection by inducing TI in Mφ through MyD88-dependent cascade conduction (235). These protective effects are partly mediated by metabolic reprogramming, characterized by sustained enhanced glycolysis, mitochondrial oxygen depletion (236, 237), and mTOR activation (235).

Interestingly, a recent study has demonstrated that mevalonate (MVA), a metabolite in the cholesterol synthesis pathway, can induce TI *via* activating the IGF1-R and mTOR pathways and subsequent histone modifications in Mo and Mφ (167) (**Figure 3**). In MVA-trained Mo, cellular metabolism switches from OXPHOS to aerobic glycolysis, and the enhanced glycolytic pathway also promotes the MVA metabolic pathway (140). Mevalonate can induce the enrichment of H3K4me3 on the IL-6 and TNF-α promoters (141), thereby enhancing the production of inflammatory factors. It is well-known that the enhanced glycolytic pathways favor the induction of Mφ polarization toward M1-type Mφ (238), as well as the production of various inflammatory cytokines, including IFN-γ and TNF-α. These cytokines eliminate the cellular internalized parasites by triggering the oxidative burst pathway (239). Moreover, there is accumulating evidence showing that TI can enhance innate immunity and reduce parasitemia in rodent models of *B. microti* and malaria (240–243). With the advance of TI research, it is believed that more and more metabolites with immunoregulatory activity will be identified in TI induction, which may be utilized to boost innate immunity in developing TIbV against parasitic diseases.

## Stimulation of hematopoietic stem and multipotent progenitor cells

Hematopoietic stem cells (HSCs) in the bone marrow (BM) are pluripotent and self-renewing (244). A large body of recent evidence suggests that inflammation or infection elicits an immune response in the BM (245–247). Similar to TI in Mo and Mφ, several infectious stimuli can train HSCs and progenitor cells, thereby enhancing the strength of the response to secondary infections (244). In the model of severe infection or sepsis, hematopoietic stem and multipotent progenitor cells (HSPCs) in the BM are also activated to proliferate and prompt BM hematopoiesis, facilitating infection control (246, 247). Moreover, *Plasmodium sporozoites* lead to the sustained proliferation of Sca-1 HSPCs to deal with life-threatening infections (248). Studies have demonstrated that mice treated with *Fasciola hepatica*-excretory/secretory (Fh-ES) can imprint a lasting memory on HSCs in the BM through metabolic and transcriptional rewiring (249). Treating mice with Fh-ES has enhanced the expansion and proliferation of myeloid-committed precursors, which leads to the expansion of anti-inflammatory Mo. This helminth-induced anti-inflammatory trained immunity reduces the susceptibility of mice to experimental autoimmune encephalomyelitis (250). Recent studies have reported that HSPCs develop trained immunity after stimulation with β-glucan or BCG. In detail, BM stimulated by β-glucan induces expansion of CD41-biased HSCs and BM subpopulations, followed by enhanced metabolic pathways such as glycolysis, cholesterol synthesis, and mevalonate in HSPCs (251). The first exposure of BM to β-glucan increased the intensity of the response of HSPCs to secondary LPS infection by expanding lineage Sca-1c-Kit (LSK) and multipotent progenitors (MPPs) and by enhancing the DNA damage response (251). BCG trains HSCs and MPPs in the BM through an IFN-γ-mediated pathway, causing cell expansion and enhancing BM hematopoiesis (187). These trained HSCs gave rise to epigenetically modified Mφ, more resistant to *Mycobacterium tuberculosis* infection (187). Thus, trained HSPCs enhance the body’s resistance to infection, and the mature immune cells generated by their differentiation have a more robust response strength to secondary infections, ultimately improving the body’s defense against pathogenic microorganisms. Therefore, the better utilization of myeloid cells in future parasite vaccine development would have the potential to dramatically improve vaccine efficacy, which would help break the cycle of parasite transmission.

Myeloid-derived suppressor cells (MDSCs) arise from myeloid progenitor cells (252), which are a population of myeloid cells that arise in a range of pathological conditions such as cancer (253). These cells are divided into two main cell types: granulocytic (G-MDSC) and monocytic (M-MDSC) (253). MDSCs are powerful immunosuppressive cells (254), capable of suppressing the function of T cells and becoming regulators of the body’s immune function in many pathological situations (253). In *S. japonicum*-infected mice, G-MDSC was found to inhibit Tfh cell proliferation, possibly through the programmed cell death protein 1 (PD-1)/programmed cell death ligand 1 (PD-L1) pathway (255). In controlled human malaria infection (CHMI) experiments, a sustained increase in circulating G-MDSC was found in volunteers who developed *Plasmodium falciparum* parasitemia, which suppressed the proliferation of CD4^+^ and CD8^+^ T cells and led to a decrease of lymphocytes, interfering with the production of immune memory against the parasite and suppressing the body’s immune response (256). In *T. cruzi*-infected mice, expansion of iNOS-expressing MDSCs had an immunosuppressive effect. Expansion of MDSCs associated with the depletion of L-arg by Arg1 impeded nitric oxide production, further exacerbating immunosuppression in mice (257). Overall, MDSCs proliferate abnormally and exert immunosuppressive effects through the mechanisms described above during chronic parasite infections (255–257), which can significantly limit the effectiveness of parasite vaccines.

Interestingly, foreign agents like β-glucan can modulate this suppressive cell population in tumor models (258). *In vivo* oral administration of particulate β-glucan significantly reduced tumor weight and splenomegaly in tumor-bearing mice (259). Differentiated M-MDSC by β-glucan *in vitro* can induce antigen-specific Th1 and CD8^+^ T cell responses *via* interacting with dectin-1. Moreover, the M-MDSC stimulated by particulate β-glucan can differentiate into CD11c cells with high MHC class II expression *in vivo*, thereby reducing tumor growth (259). Another study reported that treatment with particulate β-glucan substantially reduces MDSCs in tumor-bearing mice but increases the infiltrated Mφ and DCs, thus inducing Th1 and CTL responses and suppressing the immunosuppressive effects of regulatory T cells (260). Overall, the discoveries in tumor models may offer the possibility of enhancing host defense against parasitic infection by β-glucan inhibiting the immunosuppressive functions of MDSCs.

## Summary and perspectives

Because of the complex life history and large genome of parasites, it is difficult to find suitable parasite antigen molecules for effective vaccine development. Moreover, the parasite and some of its components can suppress the body’s immunity against infection, thus reducing the effectiveness of parasite vaccines. Interestingly, the description of TI has attracted extensive attention, representing a more effective innate immune response against the same or unrelated stimuli after a primary stimulation (94, 95). Therefore, enhancing the innate immune responses based on TI provides a unique perspective for parasite vaccine development. Enhanced innate immunity facilitates the killing of newly invading larvae at an early stage and substantially reduces the parasite burden on the organism to avoid parasite-induced type 2 immunity at a later stage (48, 69, 70). Therefore, there are promising directions for using TI to enhance the intrinsic immune response to improve the protection rate of parasite vaccines.

However, it should be pointed out that the progress of TI research is still in its infancy and faces important challenges. Research on the function and mechanism of TI in parasitic infections is just beginning, which will limit the rapid progress and practical application of TIbV. Due to the differences in immune evasion modalities of protozoan parasites and helminths (48–80), controlling parasitic infections through a strategy of TI against the same or unrelated stimuli after the initial stimulation (94, 95), is not a foolproof way. This results from the fact that parasites parasitizing different biological sites in hosts may trigger various immune responses (261). Moreover, helminth-mediated type 2 response is not always bad for hosts. For example, a short initial Th2 response is beneficial to worm expulsion, while a prolonged activation of the type 2 immune response may lead to a shift in the T-cell pool, thereby increasing the number of Treg cells (262). This hinders the over-activation of the immune system and consequently, limits the excessive collateral organismal tissue damage in chronic inflammation. Thus, how to keep the balance of type 1/type 2 response in the development of TIbV against parasitic infection, is still a big problem.

Furthermore, the future applicability of TIbV needs to be carefully considered due to the diverse disease spectrum in different regions. TIbV utilization may be more appropriate in the areas popular with parasitic and infectious diseases; however, it may not be suited in developed countries where non-communicable diseases are predominantly prevalent (263). Trained immunity can also have deleterious systemic consequences, as it may trigger enhanced tissue damage in chronic inflammatory states (95), such as increased susceptibility to atherosclerosis in patients with autoimmune or chronic inflammatory diseases (*e.g.*, rheumatoid arthritis) (264). Moreover, western diets are reported to trigger maladaptive TI, an immune state that may be responsible for common inflammatory diseases (*e.g.*, type 2 diabetes or Alzheimer’s disease) in developed countries (95). In addition, prolonged or excessive inflammatory responses can promote tumor progression (42). Overall, there are numerous challenges that need to be addressed before the clinical application of parasitic TIbV.

## Author contributions

WP and DW conceived the manuscript. JZ, JL, and CY wrote the manuscript. All authors contributed to the article and approved the submitted version.
